# A Study on the Life Experiences of Adolescents Who Grew up with Younger Siblings with Developmental Disabilities: Focusing on Phenomenological Analysis Methods

**DOI:** 10.3390/brainsci11060798

**Published:** 2021-06-17

**Authors:** Sunwon Park, Wonjung Ryu, Hyerin Yang

**Affiliations:** Center for Social Welfare Research, Yonsei University, Seoul 03722, Korea; swsailor@hanmail.net (S.P.); rinyang0103@gmail.com (H.Y.)

**Keywords:** developmental disabilities, siblings of a person with disability, qualitative research, phenomenological analysis methods

## Abstract

This study aims to explore specific life experiences and what it means to “live as a sibling of a disabled person”, by focusing on the brothers and sisters of persons with disabilities; this is a cohort that has been relatively marginalized in the field of welfare for the disabled. To this end, the author conducted 1:1 in-depth interviews with four adolescents who grew up with younger siblings who have developmental disabilities, and analyzed the meaning underlying their life experiences through phenomenological research methods. As a result, a total of five core themes of those life experiences were identified: (1) the birth of a disabled younger sibling, wherein their trials began; (2) surviving differentiation within the family; (3) ambivalence toward parents; (4) adolescence, with resurfaced psychological conflicts and relieving emotions; and (5) a future to be planned around a life of coexisting with disabled siblings. This study aims to provide basic data for social welfare intervention through an illuminating and deeper understanding of the lives of siblings of the developmentally disabled who require a high level of care.

## 1. Introduction

Unlike other disorders, developmental disabilities occur relatively early in life, and are characterized as chronic [1,2]. Accordingly, family members of persons with developmental disabilities are handed long-term roles in caring for their special needs, and siblings are not exempt from this. 

In Korea, with the enactment of the Act on Welfare of Persons with Disabilities in 1981, various rehabilitation and welfare programs were developed for persons with disabilities. As the need to protect not only the disabled individual but also their family has expanded, support systems for the family have been developed, such as the Family Support Center. Unfortunately, however, family support services and programs in Korea have focused mainly on parents, reflecting relatively little interest in the siblings of persons with disabilities [3,4]. Although the number of adolescent siblings of persons with developmental disabilities is estimated to be about 37,000 in Korea, the role of siblings in the family has been underestimated and studies on them have been insufficient [2] (p. 1).

In general, sibling relationships are the basis of cognitive and emotional development [5], and are important in providing a context for social development [6]. Furthermore, sibling relationships go through an evolutionary process throughout their lives, especially in adolescence [7], as the children begin to establish their own identities inside and outside their families; intimacy between siblings tends to increase [8].

However, the limitations of a child with disability exposes their siblings to internal and external tensions over a long time period [9], and they have different roles and experiences than those in ordinary families [10]. Nevertheless, siblings in families with a disabled member not only provide emotional support that cannot be provided by their parents [11], they also act as a proxy for caretakers, and play a special role in the family system [6] (p. 1). Therefore, it is important that siblings of persons with disabilities grow up with support, psychologically and socially, for healthy family development [12,13].

According to related studies, children’s experiences because of their disabled siblings vary greatly. They face a huge turning point with the birth of a disabled younger sibling. In the process of accepting and adapting to their younger siblings’ disabilities, they feel a great deal of resistance and tension. In fact, from childhood, they repeatedly experience alienation and differentiation through the concentrated interest toward their disabled siblings [14], and in this environment, they experience negative emotions, such as lack of affection and frustration of desire [15]. They are conflicted between their own needs and their disabled siblings’ needs as they grow and develop, such as how they feel about burdened interactions with their disabled siblings [16,17,18]. In some cases, they feel psychological pressure from the excessive responsibilities and restraints that come with caring for siblings [19,20]. In addition, they still encounter prejudices and discriminatory views towards disabled persons and their family members from Korean youth [21], therefore they perceive their own family as being inferior to other families [22].

Western studies with different cultural contexts have also found that early adolescents with siblings with developmental disabilities experience negative behaviors more often. The more time the siblings spend together, the higher the likelihood and risk of conflict, and the lower the level of well-being [23,24].

What should not be overlooked, however, is that those with disabled siblings do not only have negative experiences. They encounter special opportunities with their disabled siblings. Several studies have found that adolescents with disabled siblings can enjoy a number of “positive experiences”, such as warm contact or affectionate exchanges with their siblings that stem from observing the growing process of their sibling with disability [25], [19] (p. 2). In addition, through their experiences, they may not only develop selflessness and caring tendencies toward others [1] (p. 1) but also love and gratitude for their presence [2] (p. 1). At the same time, they may also develop positive self-awareness, insight, persistence, and more [26].

As such, siblings of persons with disabilities experience psychosocial growth as well as crises within the context of their families, thereby responding to the environment in which they live their own lives. Most of the Korean and international studies conducted so far, though, have focused mainly on adult siblings or on parents’ reporting their children’s problems, making it difficult to confirm the specific life experiences of adolescent siblings [27]. In addition, their experiences tend to have been only partially verified with a structured quantitative tool [28], and have failed to consider specific contextual factors. Siblings of persons with disabilities are important family members. In order to provide them with effective support, it is important to review their life processes through their own vivid voices. Therefore, this study aims not only to illuminate the lives of siblings of persons with disabilities but also to expand related discussions by examining the specific life process of “living as siblings of persons with disabilities” through qualitative interviews.

To achieve these ends, this study applied a phenomenological research method [29], which describes the common meaning of participants’ lived experiences to reveal their individual meanings behind growing up with younger disabled siblings. By applying the phenomenological research method [30], meaningful statements expressed through their voices were described directly, with semantic groups derived from those statements developed into subjects. Finally, an analysis process converted these into academic terms. The intention is to help people understand the lives of non-disabled brothers and sisters growing up with their developmentally disabled siblings.

## 2. Materials and Methods

### 2.1. Research Participants

Table 1 presents the general characteristics of the participants. This study was conducted with adolescents of middle- and high-school age in Korea who were judged to be able to understand the aim and purpose of this study. A total of 4 adolescents—2 males and 2 females—with younger siblings who have developmental disabilities participated in this study. All of the participating adolescents reside in two-parent households (father and mother). The age difference from their disabled siblings ranged from 2 to 9 years. The majority of the participants were not aware of the specific type of disability in their siblings. 

### 2.2. Data Collection

This study is about 4 adolescents’ life experiences with their developmentally disabled siblings. Recruitment was carried out through convenient sampling with the help of related organizations, such as the Korea Welfare Center for families with disability and the Korea Rehabilitation Support Center for Children with Disabilities, which provided the study participants with easy access for the interviews. Initially, a total of 6 adolescents participated in qualitative interviews. However, in such an interview process, they accordingly discovered the different degrees of their acceptance of the disabilities, feelings toward their siblings, and subsequent life experiences, which varied, dependent on whether their disabled siblings were older or younger. Thus, this article is only focused on “adolescents with younger siblings with disabilities.” Furthermore, as this study adopted a phenomenological approach [31] in order to understand the common experiences of individuals, one participant, who was too far out of the common experience from the other participants, was excluded.

The interview to collect qualitative data lasted around 1 to 1.5 h per interview, centering on 1:1 in-depth conversation. The researcher met with each interviewee at least 2 and up to 4 times, depending on the condition of the participant such as motivation to participate, amount of experience related to siblings with disabilities. Interviews were held in an independent space, including a counseling room, a conference room, a restroom in the facility, and a participant’s house. Qualitative interviews and data collection were conducted from August 2019 to February 2020. The content of the in-depth interview was used as the main data for analysis, but as this study employed a phenomenological analysis method that vividly reveals the world of everyday experiences, the observations derived from the interview process were based on data and information gathered from social workers, parents, teachers, and other family members, which were used comprehensively [32].

The specific procedure for the qualitative interviews was as follows: before each interview, there was a brief introduction between the participants and the interviewer, at which time the interviewer explained the aim and purpose of this study to them; in addition, participants were informed of the expected benefits and risks of this study as well as the methods being applied to their data, and finally, the procedure for obtaining participants’ consent was completed. The author conducted the interviews. The other researchers were experts with more than 10 years of practical experience in welfare fields who were also parents of children with disabilities; they each brought a deep understanding of the environmental context for families with disabled persons. However, we were cognizant of the risk that the personal, biased experiences of the researchers could involve in this study, thus, the data analysis was cross-checked with professional researcher who could “distance” themselves, providing an objective view.

Prior to delving into the subject of the interview, the interviewer spent time speaking about everyday topics with the participating youth, to form a rapport. Questions focused on those that could lead to the structural and organizational skills of the experience [31] (p. 3). We progressed to more specific questions such as “What did you experience while growing up with your sibling?”, “What does a sibling with a disability mean to you?”, and “What is the most memorable experience among the related experiences?” In addition, the order of questions was flexible, and changed according to the interview situation. Transcripts were written up within 2–3 days following an interview, and any non-verbal factors such as facial expressions and tone of voice that were identified during the interview transcription were also recorded. Private information such as name and address, institution name, school, etc., were marked with initials to protect the participating youth. This study is based on the Bioethics and Safety Act with an IRB review to ensure the protection of the rights of participants, securing bioethics and safety (Institute Review Board (IRB) of Yonsei University, approval number: 7001988-201809-HR-298-04), obtained before this study was conducted.

### 2.3. Data Analysis

This study conducted a phenomenological analysis to deeply explore the life process of adolescents with developmentally disabled younger siblings. The main purpose was to closely grasp their experiential meanings as well as capture their own worlds of experience, such as what and how they felt in the process of growing up with their disabled siblings, and what this life process meant for them. In that sense, it was judged that a phenomenological research method [33,34] was appropriate, in that it targets the work of vividly revealing the structure of experience and its related meanings. In fact, phenomenological research has not been established as a single theoretical system due to many scholars developing it in a variety of ways, so there is no standardized framework [35]. However, there is common agreement that it describes the common experiential meaning of individuals or groups for a specific phenomenon or situation [31] (p. 3). Thus, this study attempted to understand vivid experiential meanings based on the experiences of adolescents who have younger disabled siblings.

More specifically, the phenomenological analysis procedure of [30] (p. 2) was used, with a categorial analysis process added to classify the relationships among concepts, meanings, and subjects according to logical relevance. The first step was to read the transcript several times and go through a “general recognition” stage [30] (p. 2), focusing on the common experiences of the participants [34] (p. 4). After that, the author identified statements directly related to the experience of “living with a younger sibling with a developmental disability”. Specific concepts and meanings were identified through the step of deriving a “semantic unit” [30] (p. 2). Afterwards, common themes were collected according to the relevance of the identified meanings. For the third step, categorized semantic units were transformed into terms that are easy for readers to understand and described accordingly [31] (p. 2). In this process, a total of 138 semantic units and 10 main themes were identified. In the last step, we cross-reviewed them, one by one, through the process of discussion and feedback, and went back to the original data with the derived data to review whether the original meaning stated by the participant was properly reflected. Ultimately, the qualitative analysis data reviewed through this series of processes consisted of a total of 5 central themes and 9 semantic units. In this study, core categories and topics were arranged in a temporal, stepwise manner, starting with the “birth of a disabled sibling”. Subsequently, with the addition of other types of data, such as observations and program journals―especially those obtained from parents—the author has tried to broaden the understanding of their life processes.

## 3. Results

The purpose of this study is to understand the life experiences of adolescents who have grown up with a disabled younger sibling, in a disabled family; the derived components are shown in Table 2.

### 3.1. Birth of a Disabled Younger Sibling and the Trials That Began; Anxiety That Began with Awareness of a Disability

#### 3.1.1. Early Grades of Elementary School; Disability Recognition

It was found that none of the participants had ever heard directly, and in person, about their younger sibling’s disabilities. They said that they learned about the disability indirectly, when they heard their friends’ conversing about their siblings’ differences, or they became aware of it in the process of their growing up by accidentally listening to conversations between adult family members. It was also found that they had never asked their parents about their siblings’ disabilities first, and they said that bringing out the word “disability” itself seemed to hurt their parents, who were enduring the situation at the time. As such, even at a young age, they sensed the heavy family atmosphere, paying attention to their words and actions. The lack of opportunity to communicate and understand the disability of the younger sibling with the parents can partially explain the fact that the majority of them did not know the exact disability information, such as the disability type and disability level of their siblings. On the other hand, when they became aware of the disability of their younger siblings, they were at least 6 years old, and up to grades 1–2 in elementary school.

“I overheard my grandmother and mother’s conversation. After that, I never asked [about my sibling’s disability]. There is something like that, you know. I’m afraid it will hurt my mom’s heart”. (Participant C)

“Some of my friends at the playground said that your little sibling is a little weird. Come to think of it, yes, my sister is a little different. It was like this” (Participant B).

“My parents have never given me any information about why my younger sibling is sick, how long he will be sick, and what disability grade he is given. Naturally, I didn’t ask about my sibling’s pain. It wasn’t that anyone told me not to ask, but I felt like I shouldn’t ask. Even though I was young, I think it was like that”.(Participant A)

#### 3.1.2. Overwhelming Fear Due to Irrational Beliefs

Regardless of how they learned about their sibling’s disability, their perception of the disability was found to be the starting point for feeling various forms of difficult emotions, such as fear, compassion, and pity (Participant B, D). They said they were obsessed with irrational convictions, especially as their recognition of the disability was mainly achieved during their lower grades of elementary school. For example, Participant 1 felt guilty for the fact that the disability had developed in her sister instead of herself while feeling compassion and pity for her disabled sister. It turned out that Participant D felt fear because of the irrational belief that she could be abandoned by her parents. From Piaget’s theory of cognitive development, the first time they perceived a disability was during the “concrete manipulation” stage, in which their logical reasoning ability was so simple―an unstable period―that only thoughts about objects distinctly seen are possible. From this point of view, their irrational beliefs are interpreted as a phenomenon that could appear naturally in the process of immature cognitive development. Unfortunately, parents could not focus sufficiently to correct their children’s irrational beliefs or measure their anxiety. It is believed that the lack of supportive resources around their environment amplified their anxiety.

“I’m rather okay now, but when I first learned that my sibling had a disability, I was most afraid. That was when I was in elementary school, when I was in the first grade. I thought, what if I get sent away? All these terrifying thoughts…” (Participant D)

“Isn’t the gene that should have come to me went to my younger sibling, unfortunately? So, I thought like this, after I learned about the disorder. There was a time like this... I was overwhelmed with strange imaginings. But no one can explain or tell me that it’s not. Of course, it was my fault that I didn’t ask. Because everything was focused on my younger sibling, it’s probably a luxury for mom and dad to look into my feelings and thoughts...” (Participant B)

### 3.2. Surviving in Differentiation within the Family

#### 3.2.1. Resisting Anxiety: Regression Behavior; Non-Acceptance of Disability

##### Isn’t It a Lie? You Are Normal

After being aware of their sibling’s disability, each of the participants fought the anxiety they felt in their own way. Some participants made trouble by faking illness to get their parents’ attention; some showed regressive behavior, such as urinating on a blanket or refusing to attend school. Participant C said that this behavior was a survival instinct. Participant A said that he did not want to admit his sibling’s disability, so he forced his sibling to use normal words and actions for quite some time, pinching and hitting him. When the participants grew to adolescence, they said that they felt guilty or felt sorry for their parents and younger siblings while recalling their childhood behaviors when they were unable to adapt to their younger siblings’ disabilities. As such, the past experiences were still entrenched in their thoughts and feelings as they became adolescents.

“All of my sister’s things start to bother my eyes, and I’m sick of it because I’m not mature, so I was struggling with my mother. It seems to have been a survival instinct back then. When I think about it, I was really too immature and it now hurts my heart that I made it harder for my mother”. (Participant C)

“I hate to admit that my younger sibling is sick, so I did like this: ‘You can do this too, Go ahead, Put it up and take it off,’ forcibly in this way...” (Information from parents: Participant A was said to exhibit regressive behavior for a while, such as peeing on the blanket and refusal to go to school, when the participant felt a craving for their parents’ attention due to a disabled younger sibling.) (Participant A)

#### 3.2.2. Compromising in Uneasy Situations

##### I Am an Irreplaceable Child

In the midst of responding with resistance in uneasy situations, the reaction that emerged was to “compromise”.

The participants were found to have been striving to further develop their abilities to be differentiated from their disabled siblings, such as being charming to family members, achieving high academic performance, and helping their parents. Adolescent B said that whenever a depressing atmosphere was detected in the house, in order to reinforce his presence in a differentiating environment, he took charge as the “mood-maker” for the family, to make things fun; Adolescent C said that she made a constant effort to achieve high academic performance. Each youth also reported to their parents about their disabled siblings’ health status by taking care of or observing their disabled younger siblings when the parents were doing housework, which they said was a very immediate way to receive recognition and affection from parents. One interesting thing is that in the early days, there was a strong tendency to show these features “intentionally”. However, gradually, as they took them on, they started to perceive them as “their roles”. In other words, they deliberately tried to be irreplaceable in order to survive as children, but as such actions were repeated over time, they took on a sense of responsibility for the role in itself.

“I tried to make (my parents) laugh. I thought that was what I could do well in my family, and every day my mom and dad said, ‘We wouldn’t have done it without my darling ** (participant name).’ In such a case, I am proud of filling in for my younger sibling R’s shortcomings... (laughs)” (Participant B)

“I felt like I have been too mature ever since my childhood, and I kept doing it to my disabled sibling [jealousy] and then I thought that I should overcome it with studying. I thought I should change my direction. That was also something I could do to please my mother. (Information from the Participant’s family member: The Participant’s grades were the highest in the school.) (Participant C)

“It looks like a strategy, but in front of my parents, I acted like I cared about (my younger sibling), and if (my younger sibling was) in a strange state, I let my mother know, and then I was praised for doing that. Now it’s my job to do―purely caring for my younger sibling”. (Participant A)

### 3.3. Ambivalence Feelings toward Parents

#### 3.3.1. Sadness over Parents’ Differentiating Parenting Method

##### Why Are You Always Telling Me to Give Way?

For the majority of families with disabilities, even if parents try to give the same attention to all children, they are bound to concentrate on their disabled child(ren) due to limits in their own energy. In discussing this, participants answered that although they understand their parents’ positions, they cannot help feeling sadness and grief. From a relatively young age, participants recognized that their parents were more immersed in their disabled siblings than them, and it turned out that they experienced psychological conflicts, such as lack of affection and frustration of their desires. This psychological problem appeared to be the main cause of relational conflicts with parents.

“Even though I understood my parents’ different attitude toward me, I think I was often upset because my parents always seemed to force me to make concessions. Well, I think concession has become a habit for me now. I felt discrimination when I was too young, and that was a natural situation”. (Participant A)

“Because we are both children of our mom, I want my mom to be fair, but... Honestly, when she only favored my younger sibling unconditionally…, because of that, my mother and I got really bad last time”. (Participants D)

#### 3.3.2. Compassion for Parental Sacrifice

##### Another Burden―My Poor Parents

As mentioned earlier, the participants indicated they were angry at their parents’ discriminatory attitudes, but at the same time they felt regret and compassion for their parents who were sacrificing for their disabled siblings. Participant C said that she became depressed and upset just by looking at her parents, who had given up many things after taking care of her disabled sibling for a long time. She said she tries to provide her parents with positive and bright energy, with the idea that she should make those parents happy, but sometimes it feels overwhelming to have to raise her parents’ mood. Participant B also said that there are times when she feels a different burden toward her parents, namely, a pressure to make her parents happy on behalf of her disabled sibling.

“When I see my mom, who has been struggling for years because of my younger sibling, I feel it’s too hard emotionally, and whenever I think of my mom, I cry. As a woman, she is pitiable. I try to make her really happy, and that’s why I feel burdened sometimes”. (Participants C)

“There are times when not only my disabled sibling but also my parents feel like a burden to me. I felt burdened with the idea that I should succeed quickly to make my parents happy”. (Participant B)

### 3.4. Adolescence: Resolving Mental Conflicts and Emotions That Resurfaced

#### 3.4.1. Adolescence―Confronting Emerging Conflicts

##### That’s Right, My Handicapped Sibling Was the One Who Gave Me Sorrow

As they entered adolescence, psychological conflicts arose once again, caused by past wounds and unresolved feelings. In the case of Participant B, who identified himself as now being in adolescence, he regretted that all his family members were only interested in his disabled sibling, and he himself said that this feeling was new. According to Participant C, she said that, with a disabled younger sibling, the experience of differentiation is a daily event in the growth process, but it seems that subsequent emotions are now being expressed strongly, with the advent of adolescence. In addition, she said that it was in her adolescence that she first realized that the existence of her “disabled sibling” was causing her sorrow. Siblings of disabled persons routinely experience differentiating experiences as they grow up, and at the same time, they feel negative emotions toward their disabled siblings, and they grow up being suppressed by internal and external factors. The interview discussions revealed that they encountered their own oppressed emotions due to the ups and downs of adolescence, and the expansion of their contextual outlook during the later development process. Failure to adequately respond to these unexpected situations was found to be expressed as conflict and friction with parents.

“Recently, as my feelings of oppression burst out, I felt that the existence of my disabled younger sibling was a factor that made me incredibly pain-filled and sad. I used to think that, even if I had a disabled sibling, it was just fine. I grew up early in the expectations of my parents. This is the (discrimination) experience that I felt every time. But this is my adolescence, as my thoughts and my emotions grow, I would (keep) bringing it back to the past, haggling with my parents for that”. (Participant B)

“These days I’m very sensitive, I’m an adolescent..., I felt that my mom and my parents were trying to overprotect my sibling. Suddenly, I can’t adapt myself to this feeling and it’s new, but I think it’s been exploding these days. This kind of regret has been accumulating since I was a child”. (Participant C)

##### Will My Future Really Be Happy?

The psychological conflict among teenagers going through adolescence was not limited to the “past” and “present” experiences, but also included in the “future”—experiences yet to come. For example, Participant D began to realistically feel the burden of caring for his disabled sibling in the future when his parents would not be there for them. Participants were found to be carrying excessive burdens of having to take over the role of parent for their siblings. They recognized that their careers, spousal choices, marriages, residences, and other choices about future life were not free, due to their disabled younger siblings. Specifically, they would think about realistic issues, such as what a future spouse would think of their sibling with disability or about them providing direct care for their disabled siblings. They were found to feel depressed about being unable to make freer choices due to their disabled younger siblings.

“Suddenly, I thought like this: ‘Can I be married one day?’ I met someone I really like, but if she comes to know that I have a disabled younger sibling, I think anyone will be slow to accept that. How long should I take care of my younger sibling if my parents cannot be there anymore for my sibling? All these thoughts”. (Participant D)

“Others just need to take care of themselves. They can choose what they want to do. But at the end of my decision, I always have my younger sibling. I want to immigrate and live in the future, but if I have to take care of my younger sibling, that’s actually impossible, isn’t it? Then, should I become a social worker or a teacher at a disabled facility? Well, I think like that, but then it also feels a little bit desperate”. (Participant B)

##### Finding the Things for Which I’m Grateful in My Daily Life; Writing a Diary

Nevertheless, they were trying to manage and overcome their negative feelings in their own ways. They were relieving their negative emotions by themselves, in such ways as “finding things that (they were grateful for) in their daily lives” and “writing a diary”. In this way, rather than expressing their uncomfortable feelings to their family members, they seemed to have resolved them. This was due to family factors such as their compassion for their parents (who provide heavy caregiving roles), their responsibility as children, and their affection for their younger siblings.

“What do I do if I come to hate my family? But, it’s my family after all. When I write all the things I was upset about in my diary, I get refreshed, and I can regain my mind. (Q. Have you ever told your parents about these feelings?) I think, for my parents it can be more upsetting, so it’s better to take care of it on my own”. (Participant C)

“Then say a thankful prayer. Oh, I can eat this meal anytime and have this warm house. And when I get sick, my parents will somehow take care of me, so no worries”. (Participant B)

### 3.5. Planning the Future for a Life Coexisting with Disabled Siblings

#### 3.5.1. Embracing My Own Life as Living with a “Disabled Sibling”

##### Disabled Sibling = My Life

Most of the participants were planning and hoping for an optimistic future even though they were experiencing a difficult past and present due to their disabled siblings. They were found to have accepted their disabled siblings as part of their lives, specifically planning for a future in which they would play the role of guardians. Participant C was making specific plans around how to care for her disabled sibling, focusing on events in the developmental process, such as after marriage, after the death of their parents, and after the birth of her children. Participant D said that the birth of a disabled sibling was a great challenge to his family but, in that process, he said he was able to learn a lot of things that adolescents from non-disabled families might not realize, so he said he is satisfied with his own life now.

“My family was struggling with my sibling. But not necessarily. We lived by helping each other more, and I think we can give warm hearts to other sick people. So, the bottom line is, I don’t think it’s unfortunate to be the elder sister for my sibling”. (Participant C)

“I think having my younger sibling is somehow my destiny. It will all have a deep meaning, and if you go and live well, you will find the answer. I don’t think I’m unlucky and unhappy. In fact, my sibling contracted a disability early, but for everyone...until we die, a lot of disability can develop anytime for anyone”. (Participant D)

#### 3.5.2. Expectations for Formal and Informal Support Systems

##### You Know, the World Is Getting Better

The participating youth also showed optimism about the future, even while feeling anxious about it. In particular, they thought that policies and support services for disabled families were developing every year, and they were convinced that, with technological progress, new treatments and support for their disabled younger siblings would be better in a future society than they are now. In addition, the presence of resources to rely on in the local community, such as close relatives and friends met at the support center, neighbors, and social workers, provide them with the power not only to sustain the present but also to imagine an optimistic future.

“In the future, the awareness of people regarding disabilities will improve. Policies and support will also improve. I believe that we will be able to live better than we do now. Recently, artificial intelligence and medical systems have also been developed, so I believe that my family will get better as I become more mature”. (Participant C)

“Even when my parents are not present in the future, my aunt or uncle will never leave me alone to take care of my disabled sibling. They will definitely help me. Won’t I be able to live better than I thought?” (Participant A)

## 4. Strengths and Limitations

This study has several strengths.

First, the results of this study can lead domestic and foreign governments and related facilities to pay attention to the grievances of the siblings of persons with disabilities, and in providing an opportunity to discuss policy and practical intervention measures for their healthy growth. In particular, Korea exhibited a remarkable lack of interest in the siblings of persons with disabilities and the corresponding need for professional services. Based on the results of this study, a detailed support plan can be established for them.

Second, some of the authors who conducted this study are actually family members of persons with disabilities, and so they have a high level of basic background knowledge and understanding about how to live with a disabled person. Thus, in the process of deriving the research results, we not only had the ability to appropriately control the confounding categories but were also able to proceed with the research with the rest of the authors being beware of the risk of over and under interpretation of the research results.

There are also several limitations to this study.

First, it was not possible to separate and analyze the gender of the siblings. Because the role expected by society differs according to gender, the reactions of brothers and sisters in a family with a disabled child can manifest in different ways [10] (p. 2). Thus, follow-up studies need to conduct detailed examinations of their growth experiences, roles or responsibilities, conflict factors, and emotions through the classification of disabled and non-disabled siblings according to gender.

Second, there is the possibility of participants exaggerating/reducing reports of their experiences and were influenced by a social adequacy bias. Since adolescence is a time when social recognition and external evaluation are important, it is possible that some sensitive interview questions about negative feelings, conflict situations, and unpleasant experiences toward their disabled siblings were answered in a socially desirable way.

Third, siblings of various backgrounds were not included. This study adopted a convenience-sampling method and recruited participants among disabled family members who use the disabled child rehabilitation center and the disabled family support center. Therefore, only participants with relatively high access to welfare services and facilities were included in this study. Accordingly, there is a limitation in that subjects who actually need services, such as children from poor or single-parent families, were excluded from these qualitative interviews. Hence, a broader understanding of the disabled siblings from various types of families are required in follow-up research.

Fourth, with four participants, the sample size was not large enough. It does not meet the requirements of Phenomenological Analysis; generalization of research results may be difficult if the sample size is small. In reality, there are considerable difficulties involved in individual researchers obtaining data on siblings of the developmentally disabled while following research ethics guidelines. Therefore, these results need to be confirmed through subsequent studies using samples of sufficient size.

## 5. Conclusions and Suggestions

This study has tried to provide basic data for social welfare intervention by seeking a deeper understanding of the lives of siblings to those with developmental disabilities. Currently, the Korean government is making efforts to include the entire family, not just the person with a disability, in its welfare policy, but there is a limitation; it has been unable to establish comprehensive services for the family unit [2] (p. 1). Moreover, professional services for siblings of the disabled are greatly lacking [2] (p. 1). In other words, the state and related facilities should be aware of the grievances and maladjustment problems faced by the brothers and sisters of the developmentally disabled, and pay careful attention to what is needed in order to support them [2] (p. 1). Therefore, this study offers practical intervention plans based on the research results.

First, the siblings’ psychosocial problems should be addressed and supported from a life-long perspective. The pain and feelings they face in their development cycles vary. According to the results of this study, in childhood, they experience emotional problems that are mainly due to differential treatment compared to their disabled siblings, but in adolescence, more realistically, they experience difficulties all the more due to situational constraints enmeshed with their disabled siblings, such as contemplating their future marriages and careers. In addition, it was found that they felt negative emotions—such as loneliness, depression, and anxiety—due to the presence of their disabled younger siblings in childhood that were not properly resolved at that time, and which have negatively affected them in adolescence. Taking these findings together, providing continuous psychological support and personalized care for them with a life-long perspective is necessary, especially as a way to ensure smoother growth for their disabled brothers and sisters.

Second, wholesome communication about their siblings’ “disability” needs to be established between parents and non-disabled children, not only for their healthy acceptance of the situation but also for their disabled siblings’ sound development. This study found that most adolescents lacked information on the disability of their siblings, and that this was influenced by the family culture, where any mention of “disability” itself had been avoided from childhood. Healthy exchanges and communication about disability among family members can play a very important role in helping adolescents to adequately understand and accept the experiences and feelings they face as siblings of a person with disability [36,37]. Therefore, establishing various support programs is a necessity for promoting healthier communication cultures within the family.

Third, there is a need for a support system through “self-help gatherings”. Since non-disabled youth lack human resources to honestly share their concerns, it is necessary to establish such a support system by activating self-help groups. A self-help group among siblings of the disabled with common experiences has the advantage of sharing coping strategies according to the situation, providing mutual support, and affecting mental and physical stability, as well as positive adolescent development [2] (p. 1). Accordingly, the Korean government and local organizations are making efforts to support self-help gatherings for siblings of the disabled, but most of them are still operated by volunteers in the private sector. Moreover, since they are operated mainly for adult siblings, there are few opportunities for adolescent siblings to participate. Thus, there is a need for local government support centers to actively aid program development, as well as operations to promote self-help gatherings.

As such, making efforts to protect a family with a disabled child based on their contextual and specific understanding will be a very important part of promoting their welfare: it is hoped that the government and related organizations will be able to lead the functional growth of the siblings of the disabled through continuous and multilateral supportive efforts.

## Figures and Tables

**Table 1:** Characteristics of study participants.

Case No.	Gender	Age	Family Relations	Disabled Siblings’ Gender	Age Difference
A	Female	15	Parents; (disabled) younger sibling	Female	−3
B	Male	17	Parents; (disabled) younger sibling	Female	−2
C	Female	17	Parents; younger sibling 1; (disabled) younger sibling 2	Male	−4
D	Male	19	Parents; younger sibling 1; (disabled) younger sibling 2	Female	−5

**Table 2:** Research results.

Subject	Component	Subcomponent
Birth of a Disabled Younger Sibling and the Trials that Began	Anxiety that began with awareness of disability	-Early grades of elementary school; disability recognition -Overwhelming fear due to irrational beliefs
Surviving in Differentiation within the Family	Resisting anxiety: regression behavior; non-acceptance of disability	-Isn’t it a lie? You are normal
	Compromising in an uneasy situation	-I am an irreplaceable child
Ambivalent Feelings toward Parents	Sadness for parents’ discriminatory parenting methods	Why are you always telling me to give way?
	Compassion for parental sacrifice	-Another burden; my poor parents
Adolescence: Resolving Mental Conflicts and Emotions that Resurfaced	Adolescence―confronting emerged conflicts	-That’s right, my handicapped sibling was the one who gave me sorrow. -Will my future really be happy?
	Resolving negative emotions on their own	“Finding the things for which I’m grateful in my daily life”, “Writing a diary”
Planning the Future for a Life Coexisting with a Disabled Sibling	Embracing my own life as living with a “disabled sibling”	Disabled sibling = My Life
	Expectations for formal and informal support systems	-You know, the world is getting better

## Data Availability

Not applicable.
